# Updates on neonatal cell and novel therapeutics: Proceedings of the Second Neonatal Cell Therapies Symposium (2024)

**DOI:** 10.1038/s41390-025-03856-x

**Published:** 2025-01-15

**Authors:** Madison C. B. Paton, Manon Benders, Remy Blatch-Williams, Elizabeth Dallimore, Adam Edwards, Ngaire Elwood, Kylie Facer, Megan Finch-Edmondson, Natasha Garrity, Adrienne Gordon, Rod W. Hunt, Graham Jenkin, Courtney A. McDonald, Justin Moore, Marcel F. Nold, Iona Novak, Himanshu Popat, Carlos Salomon, Yoshiaki Sato, Mary Tolcos, Julie A. Wixey, Tamara Yawno, Lindsay Zhou, Atul Malhotra

**Affiliations:** 1 Cerebral Palsy Alliance Research Institute, Speciality of Child and Adolescent Health, Sydney Medical School, Faculty of Medicine and Health, The University of Sydney, Sydney, NSW Australia; 2 Department of Paediatrics, Monash University, Melbourne, VIC Australia; 3 Wilhemina Children’s Hospital, Neonatology Department, Utrecht Brain Center, University Medical Centre, University Utrecht, Utrecht, The Netherlands; 4 Argenica Therapeutics LTD, Nedlands, WA Australia; 5 Perron Institute for Neurological and Translational Science, QEII Medical Centre, Nedlands, WA Australia; 6 Murdoch Children’s Research Institute, Melbourne, VIC Australia; 7 BMDI Cord Blood Bank, Melbourne, VIC Australia; 8 Parent with Lived Experience, Sydney, Australia; 9 Discipline of Obstetrics, Gynaecology, and Neonatology, The University of Sydney, Sydney, NSW Australia; 10 Monash Newborn, Monash Children’s Hospital, Melbourne, VIC Australia; 11 The Ritchie Centre, Hudson Institute of Medical Research, Melbourne, VIC Australia; 12 Department of Obstetrics and Gynaecology, Monash University, Melbourne, VIC Australia; 13 Department of Neurosurgery, Monash Health, Melbourne, VIC Australia; 14 Faculty of Medicine and Health, The University of Sydney, Sydney, NSW Australia; 15 The Children’s Hospital at Westmead, Sydney, NSW Australia; 16 NHMRC Clinical Trial Centre, University of Sydney, Camperdown, VIC Australia; 17 Exosome Biology Laboratory, University of Queensland Centre for Clinical Research, University of Queensland, Brisbane, QLD Australia; 18 Division of Neonatology, Center for Maternal-Neonatal Care, Nagoya University Hospital, Nagoya, Japan; 19 School of Health and Biomedical Sciences, RMIT University, Melbourne, VIC Australia; 20 Perinatal Research Centre, University of Queensland Centre for Clinical Research, University of Queensland, Brisbane, QLD Australia

## Abstract

**Abstract:**

Cell therapies as treatments for neonatal conditions have attracted significant research and parent interest over the last two decades. Mesenchymal stromal cells, umbilical cord blood cells and neural stem cells translate from lab, to preclinical and into clinical trials, with contributions being made from all over the world. Effective and timely translation involves frequent reflection and consultation from research-adjacent fields (i.e. cell therapies for cerebral palsy, adult neurology, companies, and regulatory bodies) as well as meaningful involvement of people with lived experience. Progress to date suggests that aligning outcome and data reporting in later phase clinical trials will support our sector, as well as involving industry partners for streamlined solutions in cell manufacturing, commercialisation and regulatory processes. Importantly, our field can also benefit from resource sharing and research collaboration in novel drug therapies, small molecules and extracellular vesicles as we attempt to bridge preclinical and clinical research. In this review, we present highlights and learnings from the second Neonatal Cell Therapies Symposium (2024), held in Sydney, Australia.

**Impact:**

- Multiple cell therapy candidates have advanced through preclinical and clinical trials in neonatology, showing promising feasibility, safety and efficacy.
- Effective and timely translation is enabled by collaboration across research-adjacent fields, commercial partnerships, harmonising research outcomes and meaningful involvement of people with lived experience.
- Progress on the potential utility of cell therapies for neonatal conditions and further translational considerations are discussed in this paper.

## Introduction

Up to 17% of all newborn babies will require transfer to a neonatal intensive care unit (NICU) or special care nursery.^1^ Over time NICU processes have improved for newborns, including mechanical ventilation, feeding supplementation, infection control, pain management and medical stabilization. Infant survival and the rate and severity of adverse neurological outcomes, including cerebral palsy, have also improved.^2^ However, translation of new therapies to further reduce the risk of neurological impairment, metabolic complications or chronic diseases is sparse compared with adult medicine. Historically, neonates have been underrepresented in research, the experimental risks have been deemed too high, and the ethics of parental proxy consent are complex.^3^ It has been documented that as a result, most newborns in NICUs are exposed to off-label and unlicensed drugs.^4^ Neonates at risk of poor neurodevelopmental and physical health outcomes require ongoing innovation, with translation of safe and effective therapies from research into care. Cell therapies are under investigation as potential solutions for these neonates, with a range of sources, types and administration strategies being pursued.

The term ‘cell therapies’ refers to a range of regenerative and non-regenerative stem, progenitor and/or immune cell biologicals, typically sourced from gestational or adult tissues. The number of clinical trials investigating cell therapies for neonatal conditions is on the rise, with a focus on treating preterm lung injury such as bronchopulmonary dysplasia (BPD) and brain injury including periventricular leukomalacia (PVL), intraventricular haemorrhage (IVH), ischaemic stroke and hypoxic ischaemic encephalopathy (HIE). Many of these trials aim to prevent permanent brain injury and reduce the severity of motor impairments like cerebral palsy. In addition, there is a burgeoning of novel therapeutic research that includes drugs, small molecules and extracellular vesicles (EVs). Clinical trials of cell and novel therapies have stemmed from hundreds of small and large animal research studies to elucidate the mechanism of action, efficacy, timing of treatment and dose. At the same time, the neonatal field has been uniquely informed by adult and pediatric research, where cell and other novel therapies have progressed from the lab through to later phase trials. However, many questions and unique challenges for neonatal cell therapy research remain.

Here, we present highlights from the second Neonatal Cell Therapies Symposium (2024), hosted in Sydney, Australia. This conference brought together international experts in science, medicine, regulatory, commercial and our lived experience community to reflect on progress, challenges and the future of our field. This paper summarises our discussions and the advances in neonatal cell therapies as well as novel therapeutics, informed by relevant paediatric and adult research. In this review, we focus on the prominent cell types that are being evaluated in preclinical and clinical translational studies for the treatment of neonatal and pediatric conditions. These include mesenchymal stromal cells (MSCs), umbilical cord blood cells (UCB) and neural stem cells (NSCs).

## Progressing mesenchymal stromal cell therapies beyond Phase I research in neonatal brain injury

MSCs are a readily available, widely researched cellular therapy with a comprehensively defined profile of cytokine and growth factor secretion.^5^ In the context of brain ischemia following injuries such as stroke and HIE, non-invasively administered MSCs have demonstrated potent immunomodulation in preclinical models.^6^ Moreover, systematic reviews of cell therapies in neonates have established the consistent safety and feasibility of administering MSCs across a range of conditions in the clinic.^7^ MSC treatment following perinatal arterial ischaemic stroke (PAIS) in particular, has gained global attention with the completion of a first-in-human Phase I (*PASSIoN Trial*) investigating intranasal MSCs in the NICU.^8^ This trial established the safety and feasibility of intranasal MSCs in ten-term neonates in the Netherlands. All ten patients displayed initial pre-Wallerian changes in the corticospinal tracts on brain MRI, but only four (40%) patients showed asymmetrical corticospinal tracts at follow-up, and two (20%) infants developed cerebral palsy. A two-year follow-up of these neonates compared with a historical cohort of 39 term-born controls with similar inclusion criteria is under review. The team is now progressing this work to a Phase II randomized controlled trial (called *i-SToP-CP*), treating *n* = 162 term neonates with PAIS or HIE (following therapeutic hypothermia). Globally, there are now more than a dozen neonatal MSC trials complete or currently underway (clinicaltrials.gov).

As more research emerges to investigate the efficacy of MSCs in neonatal cohorts, it remains critical to harmonize data collection and outcomes across studies. Aligning core data collection and outcomes will enable prospective and/or retrospective individual patient data meta-analysis (IPDMA), which is regarded as the gold standard for combining data from randomized controlled trials. This is especially important in the field of MSC research as cell source, route of administration and other population factors differ substantially across studies. Solutions are required to enable comparison of outcomes even with heterogeneity in cell properties and participant variables. Individual participant data (i.e., de-identified participant-level data) provides several advantages for meta-analysis, including allowing more exact statistical modelling for rarer outcomes and more powerful and reliable subgroup analyses to examine hypotheses about differential individual-level treatment effects.^9^ Symposium discussion highlighted the necessary role for consensus outcome reporting within neonatal cell therapy trials as one strategy to support the alignment of data collection and rapid and detailed interpretation of outcomes moving forward.

Another observation is insufficient commercial involvement in MSC research for neonatal and paediatric conditions. MSCs are manufactured in the lab and ensuring MSC production quality, consistency and validation is essential. These processes are associated with significant cost and expertise. Hence, several MSC companies have emerged in this sector who function to conduct research and drive discovery of new treatments from a commercial perspective. Our field welcomes commercial collaboration in MSC research to ensure stable product supply, enable streamlined research phases and, hopefully, regulatory success. Without a scalable industry solution, progression through Phase I to Phase III and beyond will be hampered. There is also a risk that MSC treatment will be limited to newborns in centres in developed countries with substantial laboratory and manufacturing capacity. Recently, similar off-the-shelf cell therapies have gained commercial backing and modelled successful industry partnerships. Alongside neonatal investigations, Kidswell Bio Corporation (S-Quatre Corporation) has launched a pipeline of research into dental pulp MSCs for cerebral palsy. Furthermore, we have seen a surge in research for multilineage-differentiating stress-enduring cells (called Muse cells) for neonatal conditions like HIE in Japan. Like MSCs, Muse cells are immunomodulatory, and do not require donor matching or immunosuppression. However, Muse cells are distinctly characterised by their ability to differentiate and support direct repair in damaged tissues. A clinical-grade Muse cell product (CL2020) has been in research development, with preclinical studies establishing that intravenous administration facilitates brain repair,^10^ and improves cognitive and motor functions following injury.^11^ Muse cells now have clinical safety and preliminary efficacy in improving outcomes in a range of adult neurological conditions such as traumatic spinal cord injury, amyotrophic lateral sclerosis and stroke.^12–14^ Based on these findings, a clinical trial recruiting nine neonates with moderate-to-severe HIE was conducted, demonstrating safety and tolerability with no serious adverse events during the 18-months’ follow-up.^15,16^ Randomized controlled trials are now warranted. It is crucial that the global research community collaborate and invest in efforts to ensure that high-risk newborns worldwide, including those in low- and middle-income countries, have access to stem cell therapy for research and in future standard care, when appropriate.

## Driving neural stem cell therapies towards the clinic: learnings from adult research and integrating community preferences

Stem cell experts propose that NSCs are the most promising candidates for cell replacement therapy or repair of brain injury in cerebral palsy.^17^ Like MSCs, NSCs can provide anti-inflammatory and trophic support to protect and repair injured brain tissue.^18,19^ Distinctly however, NSCs can also engraft into the damaged brain when transplanted directly and form the three main types of functional brain cells: neurons, oligodendrocytes and astrocytes.^20,21^ NSCs can be sourced from either foetal brain tissue or differentiated from embryonic or induced pluripotent stem cells,^22^ each presenting unique challenges and opportunities.

NSCs have shown promise for the treatment of various adult neurological conditions in preclinical studies.^23^ As a result, NSCs are currently being tested in clinical trials for stroke, Parkinson’s disease, multiple sclerosis and amyotrophic lateral sclerosis, with emerging evidence of the safety of some cell treatments gathered in Phase I trials, and Phase II underway/published for some indications.^24^ One key lesson learnt from adult research is that immunosuppression is required after direct neurosurgical placement to prevent immediate rejection, support long-term engraftment and improve efficacy of NSCs.^25^ The use of immunosuppression will need to be carefully considered for the translation of NSCs to younger populations (infants and children), where the capacity to impair an immune response may be altered and immunosuppression requirements are different.

Unlike many of the adult neurological conditions under investigation, the developing brain is highly plastic and amenable to therapeutic intervention;^26^ suggesting that there could be greater benefits achieved from early intervention and targeting non-degenerative conditions like perinatal brain injury and cerebral palsy. From the available preclinical evidence, NSC treatment following perinatal brain injury significantly reduces the degree of brain injury and yields functional improvements in motor and cognitive outcomes.^27^ Unfortunately to date, all preclinical studies have been performed in small animals, mainly mice or rats, with high methodological variability, and none have elucidated the requirement for neonatal immunosuppression. Regardless, the mounting body of preclinical evidence has supported the progression to Phase I clinical trials of NSCs for cerebral palsy.^18,28,29^ Whilst encouraging, these studies are highly heterogeneous and there remain substantial gaps in the research knowledge. The field requires a thorough evaluation of the safety and efficacy of NSC treatment in children with cerebral palsy through rigorously designed clinical trials. The high cost and complexity of NSC manufacturing, reduced risk appetite of clinicians, hospitals and commercial cell companies for NSC application in children/infants, as well as unclear regulatory pathways, are substantial hurdles that hamper progress. Ethical concerns about the use of NSCs, particularly those from embryonic and foetal origins, continue to be raised as a limitation for their therapeutic translation. Encouragingly however, a survey of people with lived experience of cerebral palsy and their families demonstrated support for NSC research.^30^ Importantly, this included willingness to utilise NSCs from various cell sources and undergo NSC treatment despite the need for invasive cell delivery and immune suppression. This should give researchers, clinicians and policymakers confidence that the translation of NSC treatment for cerebral palsy has community support.

Before we can confidently progress research utilising NSCs for the treatment of perinatal brain injury, there are fundamental knowledge gaps that must be addressed by the scientific community. For example, it is not known whether immunosuppression is necessary (and safe) in infants, and it is critical that preclinical findings are confirmed in large animal models. In addition, the stem cell field has rapidly progressed in the last five years with increasing confidence and knowledge of the safety and utility of induced pluripotent stem cells, making them a potentially useful source of NSCs for large-scale clinical translation.

## Advances in umbilical cord blood for neonatal brain injury

UCB is readily available at birth for routine collection and cell isolation, making it an attractive source of stem, immune and progenitor cells for therapy. Extensive preclinical testing in small and large animal models has shown that UCB-derived cells are strongly neuroprotective.^31–37^ This is mediated via anti-inflammatory, immuno-modulatory and anti-apoptotic mechanisms, as well as through growth factor support, rather than by engraftment or cell replacement.^34,36,37^ In addition, PVL, often associated with extreme preterm birth, is reduced with UCB treatment.^38^ Preclinical research also supports that early UCB therapy results in an increase in the total number of mature oligodendrocytes as well as white matter myelin density.^36,39^ These studies strongly support the case for exploring UCB therapy for perinatal brain injury.

Current clinical trial evidence from nearly 20 years of research supports the safety and efficacy of UCB cell therapy in children with cerebral palsy.^40^ However, until recently, there were limited clinical trials using UCB for neuroprotection in preterm babies and no trials in extremely preterm infants (<28 weeks’ gestation). Findings from the recently completed world-first Phase I clinical trial (*Cord-SaFe*) were presented at the Symposium.^41,42^ In summary, 23 infants born 26 weeks’ gestation (range 24-27 weeks) were reinfused with a mean (standard deviation) dose of 44.4 (18.2) x 10^6^ cells/kg autologous UCB-derived mononuclear cells/kg at 13 days of life (range 12-14) with no serious adverse events.^41^ The team is now progressing this work to a Phase II randomized controlled trial across Australia, USA, Canada and Singapore to assess the efficacy of this therapy (*Cord-Cell Trial, ACTRN12624000968572*).^43^

An important finding from the Cord-SaFe trial was that up to 30% of infants born <28 weeks’ had insufficient UCB-derived cell counts for reinfusion of the high dose of 50 ×10^6^ cells/kg, while 45% of infants <26 weeks’ had insufficient cell counts for any reinfusion.^44^ In addition, preclinical evidence has shown that repeat doses of UCB improve the long-term efficacy of UCB therapy for treating perinatal brain injury.^45^ Thus, a proportion of preterm infants will not have any, or will have insufficient autologous UCB cells for therapy and thus alternative approaches need to be investigated for this population. These include the use of allogeneic UCB sourced from public cord blood banks. A Phase 1 trial has been initiated at Monash Children’s Hospital (Melbourne, Australia) to investigate the feasibility and safety of infusing partially (minimum 4/6 HLA) matched allogeneic UCB-derived cells to extremely preterm and preterm infants (<37 weeks gestation) with confirmed significant brain injury within the first few weeks of life (called the *ALLO Trial, ACTRN12623001352695*).^46^ In addition, there is emerging evidence that ex vivo expanded UCB cells could be beneficial for treating brain injury, in addition to current haematological indications, as they possess neuroprotective and anti-inflammatory properties in vitro.^47^ Therefore, expanded UCB cells for neonatal brain injury are another future research direction for this field.

## Umbilical cord blood therapies in context: are we close to an approval for cerebral palsy?

With exciting advancements underway in UCB for neonatal brain injury, it is timely to reflect on progress of UCB research in the paediatric space. As mentioned, UCB has been under investigation as a treatment for cerebral palsy for two decades.^48^ Cerebral palsy is a clinical consequence of neonatal brain injury and is the most common physical disability in childhood. There have been several randomized controlled trials of UCB for cerebral palsy, with more than 600 children treated with UCB in clinical studies.^48^ Despite the accumulated research data, heterogeneity across research design, participants and treatment protocols has resulted in uncertainty in the efficacy of UCB for treating cerebral palsy. To address this, an international, collaborative team embarked on an IPDMA to assess the safety and efficacy of UCB for improving gross motor function in children with cerebral palsy. These findings have been presented at international conferences (full results under review),^49^ as well as at the Symposium, with results indicating that UCB is safe and provides clinically meaningful benefits for improving gross motor function in children with cerebral palsy.^50^ Whilst improved gross motor function following UCB treatment is a significant finding, UCB may also convey other benefits for children with cerebral palsy that warrant investigation.^51^ Future research should therefore be designed to gather high-quality evidence on the effect of UCB on additional outcomes that are meaningful to people with lived experience and their families.

A Phase III trial to gain regulatory approval of UCB for cerebral palsy is also needed. Programs with established expertise in later-stage clinical trials as well as compassionate access programs of UCB for cerebral palsy are well placed to drive this necessary next step (*NCT03327467, Duke University*).^52^ Utilisation of high-quality banked allogeneic unrelated donor UCB is an enabler for these programs, enhancing feasibility and offering treatment to a larger patient population. Public cord blood banks have a key role as we explore how UCB units can be utilised across research and potentially implemented as standard care for the treatment of cerebral palsy.

## The role of public banks in enabling access to umbilical cord blood for research and beyond

There are three public cord blood banks in Australia, located in Melbourne, Sydney and Brisbane, which together form the AusCord network. The AusCord banks are funded by the Federal and State governments, with funding administered via the Australian Bone Marrow Donor Registry (ABMDR). The role of the AusCord banks is to collect, process, test and store UCB as a source of stem cells for haematopoietic stem cell transplant (HSCT) for the treatment of leukaemia and other haematological diseases. Until recent years, UCB units listed on the bone marrow donor registry could not be used for any purpose other than HSCT. However, given the burgeoning interest in the use of UCB for other indications, the BMDI Cord Blood Bank in Melbourne has worked with government, ABMDR, the TGA and ethics boards to develop a process of approvals and procedures whereby, with appropriate donor consent, banked UCB can be used for non-HSCT clinical research. This has opened the potential for banked UCB units to be used for registered clinical trials for neurological and cardiac repair, cerebral palsy and other types of advanced and regenerative therapies. Due to the specific consents used over time, this approval does not currently extend to use of banked UCB units as standard of care treatment in a non-HSCT setting, but this will be further reviewed by AusCord in the future. In April 2023, AusCord banks implemented an expanded consent whereby donors consent to their banked UCB being used for any approved clinical purpose, along with the option to consent for use across any ethically-approved research and commercial manufacturing. This means that, should the treatment of new indications with UCB become standard of care, there will be no impediment to future banked UCB units being used to treat these conditions into the future. Changes in the ways publicly banked UCB can be utilised moving forward is one example of how processes are being updated in response to research developments, supporting the future of both paediatric and neonatal research.

## Getting novel neonatal therapeutics to the clinic – updates and next steps

There have only a few examples of novel therapy translation in neonatal medicine in the last decade, including antenatal glucocorticoids (administered to the mother), postnatal surfactant and caffeine. Postnatal glucocorticoids and probiotics are used in some centres and clinical circumstances, but ongoing challenges prevent widespread adoption. Generally, progress in neonatology research and novel therapies is slow and there are ongoing misconceptions of patient outcomes, with neonatal pathophysiology still being discovered. To support therapeutic innovations, our field needs to encourage generation of a body of robust evidence, comprising extensive datasets gathered in the form of observational clinical data (generating associative evidence and establishing clinical relevance), as well as in vitro and in vivo preclinical small and large animal models to generate proof-of-concept. This strategy, with accumulating research since 2008,^53–58^ has enabled the 2022 *Anakinra Pilot*. This is a Phase I/IIa dose-escalation trial in preterm infants to establish safety, feasibility and pharmacokinetics of anakinra (an inhibitor of the pro-inflammatory cytokine interleukin 1).^59^ With safety, feasibility and pharmacokinetics of anakinra now established, international multi-site Phase III trials are planned to demonstrate the efficacy of anakinra at ameliorating preterm inflammatory diseases. Following the success of anakinra, there are now several other novel therapies under investigation for fetal growth restriction (FGR) and other placental and metabolic complications in perinatology.

## Fetal growth restriction and the therapeutic potential of thyroid hormone analogues

FGR is the failure of a foetus to reach its genetic growth potential and is characterised by foetal size being smaller than anticipated at any given gestational age. FGR is commonly caused by placental insufficiency, which leads to chronic hypoxaemia and reduced nutrient supply to the foetus. FGR affects 3-9% of pregnancies in developed countries,^60^ and is a significant cause of perinatal morbidity and mortality.^61^ Babies with FGR have an increased risk of abnormal brain development,^62–65^ and long-term neurodevelopmental deficits, including cognitive, motor and anxiety disorders,^66–70^ and a 10-to-30-fold increased risk of developing cerebral palsy.^71,72^ Abnormal white matter development and delayed maturation of the FGR brain is thought to underlie these neurodevelopmental deficits. Currently, no treatment exists that corrects placental insufficiency and the resulting neurodevelopmental deficits.

Thyroid hormones are critical for normal brain development.^73^ However, the protein responsible for transporting thyroid hormones into neural cells, called monocarboxylate transporter 8 (MCT8), is reduced in the FGR brain,^74^ making thyroid hormone therapy ineffective for promoting brain development in these babies. Preliminary data from preclinical animal experiments using the MCT8-independent thyroid hormone analogue, 3,5-diiodothyropropanoic acid (DITPA), show that short-term postnatal administration of DITPA promotes oligodendrocyte maturation in newborn FGR rats, restores myelination in newborn and adolescent-equivalent FGR rats, and reduces cognitive and anxiety-like deficits in adolescent-equivalent FGR rats with sex-specific effects. Additionally, there are no adverse effects of DITPA on neonatal growth, body composition or bone strength. DITPA may therefore be a viable option for improving neurodevelopmental outcomes of FGR.^75^ Large animal research with future complimentary clinical trials should be designed to investigate this further.

## Future of nanoparticle treatments for fetal growth restriction

Many potential neuroprotective drugs have failed in research due to their inability to penetrate the blood-brain barrier. To overcome this challenge, nanoparticles that can penetrate the brain and specifically target damaged cells in the injured newborn brain are of interest.^76^ Like most neonatal brain conditions, inflammation is shown to be a key mechanism associated with impaired development.^77^ A modified formulation of degradable nanoparticles can target, and are internalised, within neurons and microglia in the injured brain.^76^ Curcumin, the active ingredient in turmeric, has potent anti-inflammatory actions and modulates microglial activity. By encapsulating curcumin with this formulation of nanoparticles there is the opportunity to directly deliver this anti-inflammatory reagent to exert specific neuroprotective effects in the FGR newborn. Preliminary findings demonstrate intranasal is a more favourable administration route than intravenous with higher uptake of these nanoparticles into the brain and visual co-localisation with neurons and microglia in the newborn FGR piglet brain 4 hours after administration.^78^ Safety and long-term efficacy studies are paramount prior to clinical translation and are currently underway.

## Extracellular vesicles in placental function and pregnancy complications

In addition to soluble mediators such as growth factors and cytokines, extracellular vesicles (EVs) have emerged as key players in intercellular communication, influencing processes including inflammation, immune responses and metabolic dysfunction, particularly in pregnancy complications.^79–85^ EVs are a heterogeneous group of vesicles, categorized into exosomes, ectosomes, and apoptotic bodies, based on their biogenesis.^86^ Over the past decade, several reviews have detailed the mechanisms of EV origin, trafficking, and release, emphasizing their complex roles in cellular communication.^86–88^

EVs have been extensively profiled across gestation, and distinct patterns in EV levels and content have been identified in various pregnancy complications, including preeclampsia,^89^ preterm birth,^90,91^ FGR,^92^ and gestational diabetes mellitus.^93–101^ Specifically, gestational diabetes mellitus is associated with elevated levels of placenta-derived EVs in maternal circulation, reflecting changes in the maternal environment.^97,101,102^ Conditions like hypoxia and high glucose increase the release of small EVs from trophoblasts, and these EVs carry altered molecular cargo that influences target cell activity.^95,97,103,104^ For example, PAPP-A, a biomarker for pregnancy complications, is downregulated in small EVs from patients with gestational diabetes mellitus, while feto-placental endothelial-derived EVs are enriched in proteins related to oxidative stress.^95,105,106^ These findings suggest that EVs play a critical role in placental dysfunction.

Recent reports indicate a significant contribution of adipose tissue-derived EVs to the total EV concentration in the blood, with roles linked to cell metabolism.^107^ In gestational diabetes mellitus pregnancies, adipose tissue-derived EVs also regulate metabolic processes. These vesicles carry proteins such as adiponectin and FABP-4, influencing metabolic activity in distant tissues, including the placenta.^82,107–111^ Proteomic analyses reveal that small EVs from gestational diabetes mellitus pregnancies are enriched in adipocyte-associated proteins, which may affect placental function and contribute to complications like macrosomia.^95,112^ EVs reflect the physiological state of their parent cells,^113–115^ with their cargo comprising proteins, lipids, and nucleic acids that offer insights into underlying cellular processes.^95,97,116,117^ Given their sensitivity to environmental changes, EVs are being explored as potential biomarkers for diagnosing, monitoring, and predicting pregnancy-related conditions.^104,118,119^

## Using extracellular vesicles as a novel therapeutic for neonatal brain injury - updates and future directions

As well as being used as biomarkers, EVs are being explored as novel therapeutics for neonatal brain injury. Human amniotic epithelial cell-derived EVs show promising potential for promoting neuroprotection and tissue repair. Recent evidence has shown that these EVs enhance macrophage phagocytosis, reduce neutrophil myeloperoxidases and directly suppress T cell proliferation, all contributing to improved lung tissue repair.^120^ We believe these mechanisms could also be beneficial for addressing preterm brain injury. Preterm brain injury is a significant contributor to neurological complications and developmental deficits in preterm infants, with the risks decreasing as gestational age increases.^121^ Preliminary findings in foetal sheep indicate that amniotic epithelial cell-derived EVs are safe and do not elicit a physiological response in the foetus.^122^ Further investigation into their neuroprotective and repair capabilities in the brain is warranted.

## Regulatory considerations for clinical translation of neonatal cell therapies

Cell therapies are regulated as biologics. Translating biologics into clinical trials for neonates involves navigating a complex landscape of global regulatory considerations from appropriate jurisdictional agencies (i.e., the United States’ Food and Drug Administration (FDA); the United Kingdom’s European Medicines Association (EMA); the Australian Therapeutic Goods Administration (TGA)). Our field is seeing a broad range of cell therapy sources as well as a spectrum of manufacturing quality. This poses unique challenges as we look to translate research. Despite regulatory and biological development challenges, there are several jurisdictionally dependent fast-track pathways available for novel biologics that address a significant unmet need. These fast-track pathways afford early regulatory support and guidance that is often coupled with accelerated assessment and approval as well as financial incentives by way of fee waivers or market exclusivity rights. Three key fast-track pathways were discussed by experts at the Symposium:

### EMA PRiority MEdicines (PRIME)

Therapies, inclusive of biologics, that address a significant unmet need where there is no current therapy are eligible for the PRIME pathway. Applications for PRIME are dependent on the size of the Sponsor (party responsible for the trial and application). Small to medium enterprises can apply for PRIME if they have demonstrated favourable preclinical data with clinical tolerability, whereas, established pharmaceutical Sponsors must demonstrate early-stage clinical proof of concept. Benefits for the PRIME pathway include a fee waiver for small to medium enterprise Sponsors, early assignment of a rapporteur, a dedicated kick-off meeting for development support, scientific advice guarantee of 40 days and accelerated assessment of 150 days (reduced from 210 days).

### FDA breakthrough therapy

Therapies, inclusive of biologics, that address a significant unmet need where there is no current therapy are eligible for the breakthrough therapy pathway. Eligibility is dependent on demonstrating preliminary clinical evidence of substantial efficacy over existing therapies. Benefits of being granted a breakthrough therapy include fast-track designation, intensive guidance on development from Phase 1 clinical trials and a commitment from the FDA to involve senior managers in the development process to offer enhanced guidance, priority action and decision-making support.

### FDA and EMA Orphan Drug Designation (ODD)

Therapies, including biologics, that demonstrate favourable preclinical evidence to reduce morbidity and mortality in an orphan disease are eligible to apply for ODD. Benefits for ODD include tax credits for clinical trials, exemption from user fees and 7 years of market exclusivity.

When considering regulatory pathways for neonatal cell therapies, there is no uniform pathway, rather, there is a framework of advocacy from both the applicant/Sponsor and regulator. These frameworks can support translation of neonatal cell therapies as research accumulates, with adherence to risk reductions built on case-by-case, evidence-based approaches.

## Reflections on best practice in community involvement for neonatal research: forming impactful partnerships with people with lived experience

Cell therapies in neonatal research cannot be conducted without a multi-disciplinary and collaborative approach that involves people with lived experience of NICU (i.e., parents and caregivers, NICU graduates and/or those with disability). During the Symposium, we discussed the ways that researchers can create meaningful and impactful research partnerships and work to build a research team that involves those with lived experience. It was evident that accessibility of research to people with lived experience remains an important factor to support involvement. In summary, there were three key points that were discussed by our authors with lived experience of NICU and disability (K.F. and N.G.) as key enablers to lived experience participation in the research process:

### Emotional support and mutual respect

Ensuring that emotional wellbeing is considered throughout the duration of the research project and in all conduct with those with lived experience is essential. Where possible, ensure that there are several members with lived experience in the research team. It was expressed that this system helps to share the emotional burden that may be felt and enables peer support. A team that includes many people with lived experience may also reduce the amount of time each member needs to contribute. Researchers often require a high-emotional demand from those with lived experience and the sharing of their very private experiences should be approached with immense respect. Involvement and open sharing of experiences to continue to improve research is an honour and something that enriches research. Researchers should avoid considering lived experience involvement as something they are entitled to and should always seek ways to improve the experience for those members with lived experience in the process.

### Utilising inclusive language

This is not only relevant to the use of inclusive language in medical terminology but also how those with lived experience are addressed generally for research purposes. An example of inclusive language is re-considering the use of the commonly used word “consumer” and the connotations that this brings. Labelling and terminology for people with lived experience in research is an area of contention and has been documented previously.^123^ Each research team should consider what terms are acceptable, appropriate and preferred for their given circumstance, and be led by their members with lived experience. Importantly, ensuring that research team members with lived experience understand the medical and scientific concepts of the research but also feel that they are equal within the team goes a long way to ensuring effective collaboration. Inclusive and appropriate language is an enabler for this.

### Consideration of time constraints

For most research team members with lived experience, research is not their full-time job. They will have life priorities and time constraints that, if not considered, could exclude many from engaging in the research process. There is an increasing importance placed on ensuring that those with lived experience are compensated for the time they give to research projects. Flexibility in meeting times and prioritising scheduling that can accommodate work and home commitments is equally important.

Moreover, ensuring that resources are available to support engagement of people with lived experience is pivotal. Whilst resources are growing, discussions during the Symposium covered how having exemplars for ways that involving those with lived experience has worked well in cell therapy and neonatal research is an important step moving forward. The Symposium also provided an opportunity for researchers and people with lived experience to reflect on research wins and positive experiences to date. The field is relatively new, and we are given an opportunity to collaborate and uphold standards for research involving people with lived experience moving forward.

## Summary and future

**Fig. 1:**
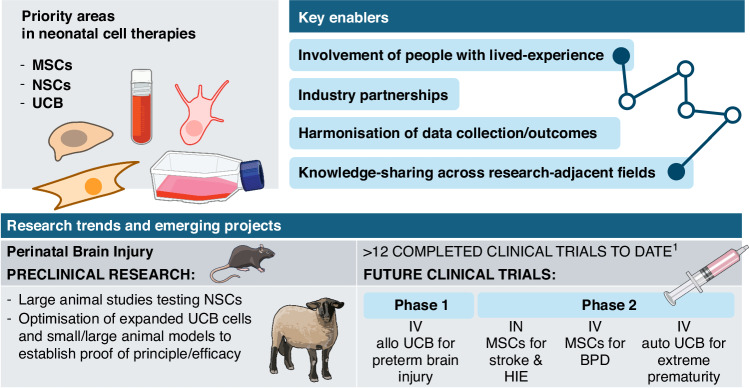
Current trends and enablers in neonatal cell and novel therapeutics. Allo allogeneic, auto autologous, HIE hypoxic ischaemic encephalopathy, IN intranasal, IV intravenous, MSCs mesenchymal stromal cells, NSCs neural stem cells, USB umbilical cord blood. Produced with NIH Bioart;^1^Clinicaltrials.gov.

Neonatal research is in a new era where MSCs, autologous and allogeneic UCB, and NSCs are reaching the clinic with several early and later phase clinical trials underway. Results of safety and early efficacy have been published and substantial progress has been made to enable ongoing and sophisticated preclinical investigations of expanded cell therapies and several other treatment candidates. Along with open and timely discussion of results, standardized preclinical study methodology and clinical trial outcome selection, we continue to critically evaluate each cell therapy treatment strategy, determine how to harmonise our work and invest in strategies that are more likely to succeed. Importantly, the neonatal cell therapies field has been enriched with positive examples of engagement with people with lived experience to deliver meaningful and transformative research. The next considerable enabler for our sector will be growth in investment and interest from commercial partners, scalable and reliable sources of cell therapies and supporting regulatory processes. A summary of the current trends and enablers are outlined in Fig. 1.

As highlighted at the previous Neonatal Cell Therapies Symposium,^124^ outcome selection within neonatal clinical trials remains challenging. It is critical to ensure that we can harmonise findings by aligning core data collection and outcomes, incorporate early outcome measures that are robust enough to detect change and setup our trials for success, especially as we emerge into a new era of sophisticated adaptive trial design. Furthermore, as referral into developmental follow-up and early evidence-based physical and occupational therapy becomes the norm, we need to ensure that these protocols are also integrated into cell and novel therapy clinical trials; cell therapies should be considered as a complement to the full picture of treatment strategies rather than as a standalone. Targeted training based rehabilitation has the potential to strengthen the new connectivity and circuity established by cell therapies. We can take many learnings from progress in research-adjacent fields including UCB therapy for cerebral palsy, adult neurology, novel therapies, small molecules and EVs.

The second Neonatal Cell Therapies Symposium (2024) provided a time to reflect on the progress of our field and future directions. We now have an array of cell and novel therapy candidates for the treatment of neonatal conditions under investigation globally. Dissemination of results, challenges and victories remains a critical component of ensuring shared success.
